# Investigating Exposure and Hazards of Micro- and Nanoplastics During Pregnancy and Early Life (AURORA Project): Protocol for an Interdisciplinary Study

**DOI:** 10.2196/63176

**Published:** 2024-10-08

**Authors:** Amanda M Durkin, Runyu Zou, Justin M Boucher, Matthew SP Boyles, Jeske van Boxel, Mariona Bustamante, Emily A Christopher, Payam Dadvand, Hanna M Dusza, Majorie van Duursen, Markus M Forsberg, Karen S Galea, Juliette Legler, Laurens DB Mandemaker, Florian Meirer, Jane Muncke, Tim S Nawrot, Petra Přibylová, Anna R Robuck, Nelly D Saenen, Barbara M Scholz-Böttcher, Kuanliang Shao, Martine Vrijheid, Douglas I Walker, Lisa Zimmermann, Laura M Zoutendijk, Virissa Lenters, Roel Vermeulen

**Affiliations:** 1 Department of Global Public Health and Bioethics Julius Center for Health Sciences and Primary Care University Medical Center Utrecht Utrecht Netherlands; 2 Division of Environmental Epidemiology Institute for Risk Assessment Sciences Utrecht University Utrecht Netherlands; 3 Food Packaging Forum Foundation Zurich Switzerland; 4 Institute of Occupational Medicine (IOM) Edinburgh United Kingdom; 5 Centre for Biomedicine and Global Health School of Applied Sciences Edinburgh Napier University Edinburgh United Kingdom; 6 Amsterdam Institute for Life and Environment, section Environmental Health and Toxicology Faculty of Science Vrije Universiteit Amsterdam Netherlands; 7 ISGlobal Barcelona Spain; 8 Universitat Pompeu Fabra Barcelona Spain; 9 Spanish Consortium for Research on Epidemiology and Public Health Madrid Spain; 10 Division of Toxicology Institute for Risk Assessment Sciences Utrecht University Utrecht Netherlands; 11 School of Pharmacy University of Eastern Finland Kuopio Finland; 12 Inorganic Chemistry and Catalysis Group Institute for Sustainable and Circular Chemistry and Debye Institute for Nanomaterials Science Utrecht University Utrecht Netherlands; 13 Centre for Environmental Sciences Hasselt University Diepenbeek Belgium; 14 Department of Public Health and Primary Care Leuven University Leuven Belgium; 15 RECETOX Faculty of Science Masaryk University Brno Czech Republic; 16 Department of Environmental Medicine and Public Health Icahn School of Medicine at Mount Sinai New York, NY United States; 17 Institute for Chemistry and Biology of the Marine Environment Carl von Ossietzky University of Oldenburg Oldenburg Germany; 18 Gangarosa Department of Environmental Health Emory University Rollins School of Public Health Atlanta, GA United States

**Keywords:** epidemiology, pregnancy, toxicology, microplastics, placenta, risk assessment

## Abstract

**Background:**

Micro- and nanoplastics (MNPs) are emerging pollutants of concern with ubiquitous presence in global ecosystems. MNPs pose potential implications for human health; however, the health impacts of MNP exposures are not yet understood. Recent evidence suggests that MNPs can cross the placental barrier, underlying the urgent need to understand their impact on reproductive health and development.

**Objective:**

The Actionable eUropean ROadmap for early-life health Risk Assessment of micro- and nanoplastics (AURORA) project will investigate MNP exposures and their biological and health effects during pregnancy and early life, which are critical periods due to heightened vulnerability to environmental stressors. The AURORA project will enhance exposure assessment capabilities for measuring MNPs, MNP-associated chemicals, and plastic additives in human tissues, including placenta and blood.

**Methods:**

In this interdisciplinary project, we will advance methods for in-depth characterization and scalable chemical analytical strategies, enabling high-resolution and large-scale toxicological, exposure assessment, and epidemiological studies. The AURORA project performs observational studies to investigate determinants and health impacts of MNPs by including 800 mother-child pairs from 2 existing birth cohorts and 110 women of reproductive age from a newly established cohort. This will be complemented by toxicological studies using a tiered-testing approach and epidemiological investigations to evaluate associations between maternal and prenatal MNP exposures and health perturbations, such as placental function, immune-inflammatory responses, oxidative stress, accelerated aging, endocrine disruption, and child growth and development. The ultimate goal of the AURORA project is to create an MNP risk assessment framework and identify the remaining knowledge gaps and priorities needed to comprehensively assess the impact of MNPs on early-life health.

**Results:**

In the first 3 years of this 5-year project (2021-2026), progress was made toward all objectives. This includes completion of recruitment and data collection for new and existing cohorts, development of analytical methodological protocols, and initiation of the toxicological tiered assessments. As of September 2024, data analysis is ongoing and results are expected to be published starting in 2025.

**Conclusions:**

As plastic pollution increases globally, it is imperative to understand the impact of MNPs on human health, particularly during vulnerable developmental stages such as early life. The contributions of the AURORA project will inform future risk assessment.

**International Registered Report Identifier (IRRID):**

DERR1-10.2196/63176

## Introduction

Plastic is pervasive in both built and natural environments. Global plastic production was estimated to be 400.3 million tons in 2022, with most plastic generated for single-use purposes [1]. Despite growing regulatory efforts to reduce plastic production and increase recycling, global plastic production is projected to increase to 1100 million tons by 2050 [2-4]. The lifetime cost of the plastics produced in 2019 alone exceeded US $3.7 trillion, including expenses such as greenhouse gas emissions, waste management, and environmental cleanup, and without accounting for potential costs related to impacts on human health [5]. Small plastic particles are generated by weathering and degradation or intentionally produced [4,6]. These particles, including fibers, are categorized based on their dimensions, with those ranging from 5 mm to 1 μm referred to as microplastics (MPs), and those smaller than 1 μm termed nanoplastics (NPs) [6]. Although there is growing evidence of micro- and nanoplastics (MNPs) in air, drinking water, and food [7], the scale of MNP exposure and behavior in the human body remains uncertain [8]. Furthermore, the potential risk MNPs pose to human health is largely unknown [9,10].

MNPs represent a complex class of pollutants, with a range of physical-chemical properties, such as morphology, composition, density, and surface chemistry. Human health risk assessment of MNPs presents unique challenges, primarily due to the complexity and diversity of these particles [9]. Established risk assessment frameworks, for example, for engineered nanomaterials and chemical pollution, are likely inadequate to account for the complex characteristics of MNPs [11,12]. Recent inventories document more than 4000 substances used in plastic packaging and more than 10,000 plastic-associated compounds, including organic polymers, additives such as plasticizers, and nonintentionally added substances such as reaction by-products [13,14]. Furthermore, MNPs in ecosystems may form eco-coronas, which may subsequently absorb other environmental pollutants and potentially facilitate human exposure to additional pollutants [15]. Current measurement approaches inadequately capture the full extent of MNP-associated chemical exposures in humans, as they focus on a limited set of known chemicals.

Various analytical techniques have provided the first insights into human exposure to MNPs. There is growing evidence that MNPs can be detected in human tissues, including blood and placenta tissue [16-18]. Mass-based assessment of MNPs using high-resolution mass spectrometry coupled with pyrolysis (Py-GC/HRMS), chemical profile assessment, and complementary spectro-microscopic characterization have emerged as promising approaches [19-21]. However, most MNP exposure assessments to date have been small scale (ie, less than 100 subjects), proof-of-concept studies, and have primarily focused on MPs rather than NPs [17,18,22]. Critical analytical advancements are necessary to reduce the uncertainty and error of exposure estimates and to enable comprehensive risk assessment [11,23].

During gestation and early life, the developing fetus is highly vulnerable to environmental stressors, including chemical exposures, due to rapid organogenesis and developmental plasticity [24]. Disruption by environmental toxicants during this window of heightened susceptibility can have a long-lasting impact on the molecular and physiological phenotype and health later in life [25]. The placenta is a unique organ at the maternal-fetal interface that facilitates gas exchange and the transport of nutrients, hormones, and other solutes essential for fetal growth and development, while also supporting maternal health [26]. It has been observed that not only endogenous but also exogenous compounds, including engineered metallic and carbonaceous nanoparticles, can cross the placenta barrier [27,28]. Growing evidence supports the placental translocation of MNPs in in vitro, in vivo, and ex vivo models [29]. Furthermore, MNPs have recently been detected in human placenta tissue, amniotic fluid, meconium, and breastmilk [18,22,30-32]. Given that pregnancy and early life are periods of heightened susceptibility to environmental stressors, evidence supporting the potential for transplacental transport of MNPs [33,34] and early-life exposure via ingestion and inhalation [35] provide impetus for investigating the implications of MNP exposure during these periods.

**Figure 1:**
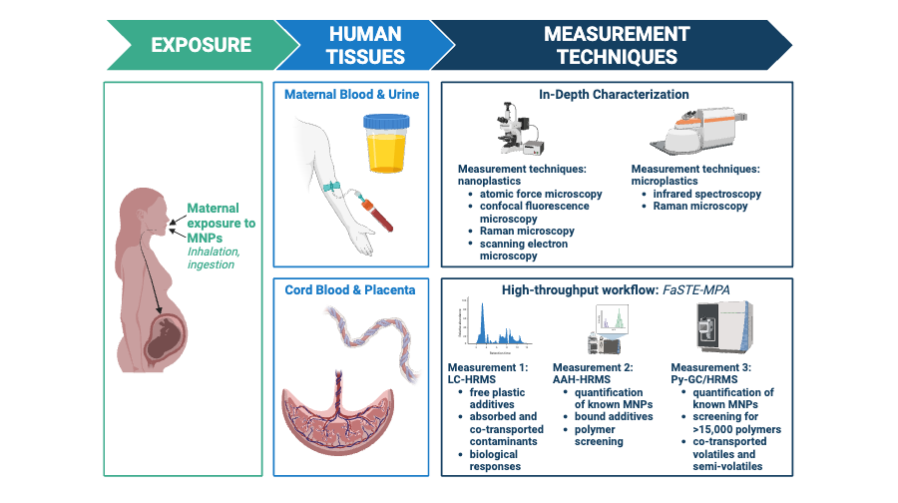
Overview of exposure assessment, sample types, and measurement techniques in AURORA. AAH-HRMS: alkaline-assisted hydrolysis with high resolution mass spectroscopy; AURORA: Actionable eUropean ROadmap for early-life health Risk Assessment of micro- and nanoplastics; FaSTE-MPA: Fast, Single, Tissue Extraction for Multiplexed Plastic Analysis; LC-HRMS: liquid chromatography with high resolution mass spectroscopy; MNP: Micro- and nanoplastic; Py-GC/HRMS: pyrolysis gas chromatography high-resolution mass spectroscopy. Image created with BioRender.com [37].

To overcome these gaps in MNP research, the Actionable eUropean ROadmap for early-life health Risk Assessment of micro- and nanoplastics (AURORA) research project aims to create a comprehensive framework for evaluating the health risks associated with MNPs during early-life stages [36]. Emphasis is placed on advancing analytical techniques for characterizing MNPs and evaluating their potential hazards during pregnancy and early life (Figure 1). This article provides an overview of the objectives, approaches, methodologies, strengths, challenges, and anticipated impacts of the AURORA project.

## Methods

The AURORA project will address fundamental knowledge gaps about the effects of MNPs on early-life health and inform future human health risk assessment [36]. This 5-year project (2021-2026) is funded by the European Union through the Horizon 2020 program and is one of the five projects that forms the CUSP European research cluster to study the health impacts of MNPs [38].

### Objectives

The AURORA project has five core research objectives:

1. Exposure characterization: to develop analytical methods for in-depth characterization of MNPs in maternal and fetal human sample matrices, including placenta.
2. Scalable exposure assessment: to develop high-throughput analytical strategies for quantitative assessment of maternal and prenatal exposure to MNPs and biomonitoring in human populations.
3. Experimental toxicology: to assess toxicity, toxicokinetics, and toxicodynamics of MNPs in experimental models, focusing on placental models.
4. Epidemiology: to evaluate associations between MNP exposure and female reproductive and early-life health outcomes.
5. Advance risk assessment: to develop an actionable European framework for human health risk assessment of MNPs.

To address these objectives, the AURORA project was designed with an interdisciplinary approach, bringing together experts in nanoparticle characterization, inorganic chemistry, analytical chemistry, exposomics, exposure science, biology, toxicology, epidemiology, risk assessment, and science communication.

### Study Populations and Samples

An extensive set of profiled human tissue samples, household samples, and biological markers and health outcomes will provide novel information about MNP exposure and health impacts. The AURORA project involves 2 richly phenotyped birth cohorts with unique biobanks, including placenta samples, and will establish a new cohort to assess determinants of MNP exposure.

The ENVIRonmental influence ON early AGEing (ENVIRONAGE) birth cohort has been recruiting pregnant women in the East Limburg Hospital, Genk, Belgium since 2010, and currently includes more than 2300 mother-child pairs [39]. This cohort was established to investigate the impact of air pollution and other environmental stressors on early biological aging. The Barcelona Life Study Cohort (BiSC) has recruited 1080 mother-child pairs residing in the Barcelona metropolitan area, Spain, during the years 2018-2021 with the overall aim of investigating the impact of the early-life exposome on maternal and child health and development [40]. Both cohorts collected fresh placenta tissue immediately after delivery. In addition to the standard biopsies sampled at fixed locations on the fetal and maternal side of the placenta [39], for a subset of women recruited since 2020, a de novo sample collection of the entire placenta was initiated with a sample collection procedure designed to minimize MNP contamination. In addition to placenta samples, cord blood, maternal urine, and blood samples from these cohorts will be analyzed to comprehensively characterize MNP exposures. To assess the impact of MNP exposures on maternal and early-life health, we will leverage the longitudinal assessments and novel health outcome data from the 2 birth cohorts, including markers of placental function, immune-inflammatory responses, oxidative stress, accelerated biological aging (telomere length), birth outcomes, and childhood growth and development.

Furthermore, a new cohort will be established in the Netherlands to assess determinants of MNP exposure, specifically among women of reproductive age (18-45 years). We will include 110 women living within 50 km of Utrecht, the Netherlands. Blood, urine, and household dust samples will be collected at baseline and after 3 months. In addition, a self-administered online questionnaire, taking approximately 15 minutes to complete, will be used to identify factors contributing to MNP exposure. This questionnaire will gather sociodemographic information, home environment characteristics, food consumption and preparation habits, as well as lifestyle and behavior patterns.

### Quality Assurance and Quality Control

One of the considerations taken across the project is minimizing both primary MNP contamination during sample collection and secondary MNP contamination during processing and analysis with quality assurance and quality control (QA/QC) measures [41]. Plastic is commonly used for collecting biological samples and has many applications in laboratory settings. We use glass sample collection materials to collect de novo blood, cord blood, and urine samples and store placenta samples in aluminum foil. Cohort samples collected with plastic materials prior to the AURORA project will be compared to samples from the de novo collection to understand background contamination, and empty collection tubes will be analyzed to determine background MNP levels. In laboratory spaces, plastic is avoided and removed where possible, and work is done under a laminar flow hood. We implement QA/QC measures including field and procedural blanks at multiple stages of collection and analysis to account for any remaining background contamination. Quality control samples are prepared by spiking samples with known MNP concentrations to monitor the accuracy and performance of the method, ensuring the quality and reliability of the data across batches.

### In-Depth Characterization of MNPs

The development of an analytical framework for the in-depth characterization of MNPs in maternal and fetal tissues is fundamental to providing measures of the particle characteristics potentially driving toxicological and health effects [9]. Key characteristics include particle quantity (mass and particle count), morphology (size, shape), chemical composition, surface chemistry, and state of degradation [19]. Low-throughput nondestructive particle-based approaches will be used for in-depth characterization.

Microscopic techniques are established for detecting and characterizing MNPs greater than ~1 μm in diameter [20]. However, additional advancements are required for the detection of NPs [20,21]. Thus, we will first focus on developing and applying innovative spectro-microscopic techniques, such as atomic force microscopy, confocal fluorescence microscopy, and scanning electron microscopy to characterize NPs [42,43]. To characterize MPs, we will leverage additional microscopy methods including Raman microscopy and infrared spectroscopy [44]. These methods will be optimized and validated using simple matrices (eg, salt and fresh water) to ensure the highest sensitivity before analyzing tissue samples.

Adding to the complexity of MNPs is that environmental MNPs undergo degradation, which makes understanding characteristics of MNPs at various stages of degradation essential for the identification of MNPs in complex matrices [45]. A vibrational spectroscopic library of MNPs from controlled degradation experiments will be established to facilitate identification of MNP type, regardless of MNP condition and state of degradation. Due to the complex nature of maternal and fetal tissues, sufficient sample digestion, filtration, and preconcentration is required to remove biological interferences prior to microspectroscopic analysis and imaging [46]. Tissue sample processing methods will be optimized to maximize sensitivity and minimize alteration of MNPs.

The spectroscopic methods for in-depth characterization of MNPs will be developed and validated with commercial polystyrene (PS) spheres. However, the advancement of toxicological models requires the availability of well-defined, comparable, and representative MNPs that span a diverse range of polymers, sizes, and shapes [47]. Synthesis of suitable MNPs is necessary due to the limited commercial availability of MNPs, which currently predominately limits research to PS spheres [48]. The synthesis of MNPs via nanoprecipitation for the most common polymer types, both with and without fluorescent tagging for detection with fluorescence microscopy, will be performed to support the development and testing of the toxicological models. These fluorescent tags are designed not to leach, ensuring they remain nontoxic.

### Scalable Exposure Assessment

The AURORA project will develop and apply a high-throughput analytical workflow to quantify MNPs in human tissues. High throughput, robust, and quantitative methods are essential for human biomonitoring and to conduct informative epidemiological studies [49,50]. We will use this approach to assess the mass concentration of MNPs and associated chemicals in maternal blood, urine, placenta, and cord blood. These metrics are chosen because they are useful for biomonitoring studies (maternal urine, blood) and suitable for early-life exposure assessment (birth cohorts: placenta and cord blood).

Our approach (termed Fast, Single, Tissue Extraction for Multiplexed Plastic Analysis: FaSTE-MPA) combines three HRMS platforms to enable systematic characterization of MNPs in complex matrices: (1) Py-GC/HRMS to characterize MNP levels and cotransported volatiles and semivolatiles, (2) alkaline-assisted hydrolysis with liquid chromatography HRMS (AAH-HRMS) to measure particle monomers and additives, and (3) untargeted liquid chromatography with HRMS (LC-HRMS) to characterize the metabolome for the presence of MNP constituents, additives, nonintentionally added substances, and biological response profiles [51,52]. Analysis by Py-GC/HRMS will provide mass concentration estimates of common plastic polymers including PS, polyvinyl chloride (PVC), polyethylene terephthalate (PET), polyamide (PA), polyethylene (PE), polypropylene (PP), polymethyl methacrylate (PMMA), and polycarbonate, as well as screen for other MNPs. Additionally, AAH-HRMS and LC-HRMS provide complementary information about both free and bounded plastic polymers and additives, such as phthalates. We will develop a comprehensive library containing the Py-GC/HRMS fingerprints of common MNP polymers and associated plastic additives. In this manner, maternal and prenatal MNP exposure and biological responses will be measured in 800 paired placenta and cord blood samples from the birth cohorts. Further, an in-depth investigation into the maternal-fetal transfer of MNPs from maternal blood to placenta to cord blood will be conducted in a subgroup of mother-child pairs, aiming to provide insight into kinetics and transfer efficiencies. Spatial distribution and accumulation of MNPs within the placenta will be assessed in a subset of placentas.

Despite the potential applications of Py-GC/HRMS for assessing MNPs, targeted Py-GC/HRMS is currently not routinely available in analytical laboratories designated for human samples. Furthermore, required sample volumes (currently >1 mL) may preclude its use in the analyses of precious biobanked samples. To identify the top chemical signals predicting MNP exposure, we will leverage untargeted HRMS data to build classification models for biomarkers exhibiting the highest sensitivity and selectivity for MNP exposure. These biomarkers will be used to establish a quantitative, targeted method that can be implemented in human biomonitoring studies (eg, Human Biomonitoring for Europe) and large-scale epidemiological studies. Results from these population-based studies are critical inputs for future etiological research and regulatory assessment of MNP exposure and health effects [10].

### Hazard Assessment: Experimental Toxicology

Despite evidence suggesting that MNPs can cross the placental barrier, placental toxicokinetics and toxicity of MNPs remain largely unexplored [33,53-55]. Knowledge gaps in human placental transfer and toxicity of MNPs hinder comprehensive hazard characterization for early-life exposure [34]. We will apply a series of toxicological models for toxicokinetic and effect studies, focusing on the placental barrier and assessing the effects of MNPs on the placenta and the developing organism. Placenta is a morphologically and functionally complex organ; therefore, placental models with increasing level of complexity, that is, monolayers, cocultures, and placental perfusion, will be used to address the complex interplay between different placental cell types [34].

**Figure 2:**
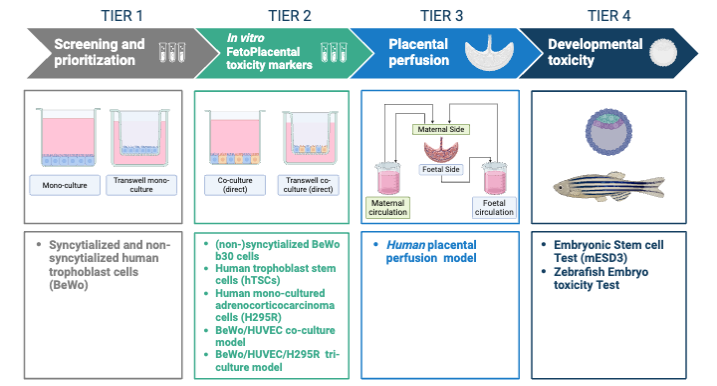
Tiered approach to investigate transport, toxicokinetics and toxicity of MNPs (micro- and nanoplastics) in toxicological models with varying complexity, including in vitro placental models, human placental models, and developmental toxicity tests. HUVEC: human umbilical vein endothelial cells. Image created with BioRender.com [37].

Considering the multitude of MNP characteristics, toxicity testing will be done in a tiered approach, using MNPs generated commercially and within AURORA (Figure 2). Many MNPs will be tested in the simple models, and based on uptake, transport and toxicity will be prioritized for further testing in more complex models. In Tier 1, toxicity end points (eg, cell viability, damage to the cell membrane, and oxidative stress) as well as uptake and transport of MNPs will be investigated in nonsyncytialized and syncytialized human choriocarcinoma cells (BeWo b30), representative of cytotrophoblasts and syncytiotrophoblasts, respectively [56]. In Tier 2, MNPs will be investigated with additional monolayer cell cultures, including the human adenocarcinoma cell line (H295R) and human trophoblast stem cells, as well as more complex coculture cellular models such as BeWo/Human umbilical vein endothelial cells (HUVEC) and BeWo/HUVEC/H295R triculture [57-59]. Tiers 1 and 2 will provide insight into a wide array of effects on placental integrity and function, immune response, endocrine functions, and other pathway perturbations. We will also perform mRNA sequencing and metabolomic analyses in selected samples to investigate system homeostasis.

Further investigations will focus on selected MNPs in a human placental perfusion model (Tier 3), the embryonic stem cell test with mouse embryonic stem cells (mES-D3; Tier 4), and the zebrafish embryo toxicity model (Tier 4). Transplacental transfer and toxicity markers will be assessed upon exposure to selected MNPs in the human placental perfusion model [60].MNP concentrations in perfusion media and tissue will be analyzed by Py-GC/HRMS and LC-HRMS analysis. Untargeted LC-HRMS and biological pathway analyses will be performed on the embryonic stem cell test and zebrafish embryos exposed to MNPs [61]. Placental and developmental toxicity markers upon MNP exposure will be compared with the health outcome assessments in the epidemiological studies.

#### Epidemiological Studies

**Figure 3:**
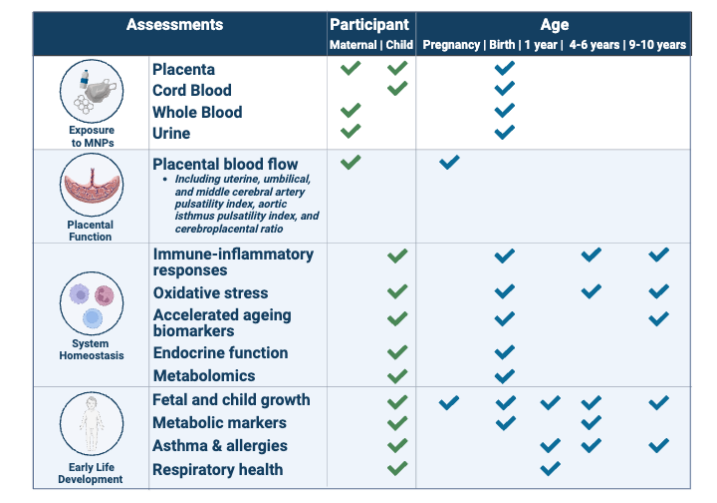
Overview of MNP (micro- and nanoplastic) exposure assessment and health outcomes to be evaluated in epidemiological studies in AURORA (Actionable eUropean ROadmap for early-life health Risk Assessment of micro- and nanoplastics). Image created with BioRender.com [37].

In epidemiological studies, we will evaluate the impact of MNPs and other plastic-related chemicals on multiple end points: (1) placental function; (2) system homeostasis; and (3) early-life growth and development (Figure 3). MNP exposure will be evaluated in 800 placenta samples from the ENVIRONAGE and BiSC cohorts. To examine the impact of (prenatal) MNP exposures on health, we will exploit the cohorts’ longitudinal health assessments to generate novel data on exposure-health associations. The children currently span from new-born to 14 years of age.

When applicable, we will pool data from the 2 birth cohorts. We will use single-exposure-outcome regression modeling adjusted for covariates, as well as complementary exposure-wide modeling for MNPs and MNP-associated chemicals, including multivariable variable selection and machine learning approaches [62-64]. In addition, we will screen for associations between MNPs and metabolomic analytes to identify the overall biological alterations associated with MNP exposure and generate novel hypotheses on potential effects of MNPs [65,66]. Moreover, mediation analyses will be performed to evaluate system homeostasis markers, using models that accommodate multiple and high dimensional mediators [67,68].

Given that MNPs are a continuous exposure variable, the minimal sample size required to detect an effect for dichotomized outcomes depends on the prevalence of the outcome. The primary dichotomized outcomes to be examined are asthma and allergy, both prevalent in 5%-15% European pediatric populations [69-71]. Due to the absence of prior knowledge on the effect size of MNPs, we referred to a study which reported an increased risk of asthma (odds ratio [OR] 1.6-3.9) in Canadian children exposed to high phthalate concentrations in house dust [72]. Assuming a baseline outcome prevalence of 5%, an OR of 2, and a type I error of 0.05, the minimal statistical power obtained with 800 participants would be 0.77, which is deemed satisfactory. The statistical power for continuous outcomes is higher given the same sample size.

Identifying the contributing factors to MNP exposure levels provides insight into how MNP exposure may influence early-life health. Therefore, we will investigate determinants of MNP exposure, including maternal food consumption, sociodemographic and other lifestyle factors in the birth cohorts. The Dutch MNP exposure cohort, consisting of 110 women of reproductive age in the Netherlands, will allow for an in-depth assessment of the determinants of MNP exposure by combining levels of MNPs in blood, urine, and household dust samples, with a questionnaire investigating MNP exposure sources. We will assess the relative contribution of food packaging, food preparation methods, and indoor sources such as furnishing and frequency of cleaning to MNP and plastic-associated chemical body burdens.

#### Risk Assessment

Risk assessment of MNPs is challenging, because MNPs constitute a very broad class of substances with diverse physiochemical properties, making it difficult to apply standard regulatory risk assessment approaches [12,73,74]. Evaluation of the exposure and hazard accounting for both the polymers and MNP-associated chemicals (ie, mixture effects) is essential [11]. Risk assessment of MNPs is also constrained by limited availability of reference materials, analytical challenges, and insufficient information about key characteristics of MNPs [11]. Further, hazard characterization is currently limited by lack of information about the accumulation, persistence, and kinetics of inhaled and ingested MNPs [10]. To understand early-life effects, risk assessment must consider the exposure rates for both the mother and the fetus.

**Figure 4:**
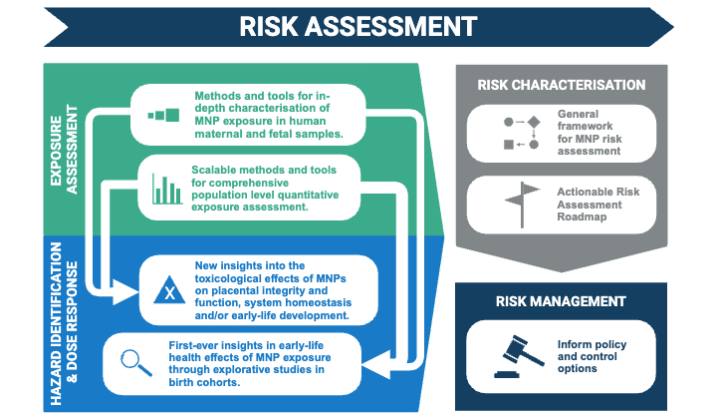
The anticipated output of the AURORA (Actionable eUropean ROadmap for early-life health Risk Assessment of micro- and nanoplastics) project, which will contribute to the development of a human health risk assessment framework for evaluating the risks associated with MNP (micro- and nanoplastic) exposure in early life. Image created with BioRender.com [37].

We will build a framework for performing human risk assessment for MNPs with a focus on direct risk to the fetus and maternal-mediated risk, including via the placenta (Figure 4). A systematic evidence mapping approach will be applied to evaluate relevant literature from organizations such as the World Health Organization, European Food Safety Authority, Organization for Economic Co-operation and Development, National Institute for Occupational Safety and Health, and the published scientific literature, and critically evaluate the available regulatory tools for their relevance and adequacy for assessing the risks of MNPs. The framework we develop will integrate the project’s updated methodologies and tools for assessing MNP exposures and risks from toxicological and epidemiological studies. Ultimately, we will determine the requirements to carry out a comprehensive risk assessment of MNPs and develop recommendations for advancing risk assessment for MNPs, focusing on early-life health.

### Ethical Considerations

The research activities of AURORA comply with international conventions and ethical codes, including the Declaration of Helsinki (2013) and the Declaration of Taipei (2016). Local ethics committees will support participating research groups to address all ethics requirements. Measures for personal data protection and confidentiality comply with the European Union General Data Protection Regulation (2016/679) and the FAIR (findable, accessible, interoperable, reusable) Data Principles [75].

The toxicological studies adhere to the 3R principles emphasizing nonanimal, in vitro cell, or alternative animal models [76]. Zebrafish fall outside the definition of animal models, eliminating the need for a specific ethical permit [77]. Human ex vivo placental perfusions with MNPs received ethics committee approval from the Research Ethics Committee of Hospital District of Northern Savo (952/2022). The ENVIRONAGE and BiSC cohorts are approved by the Ethics Committee of Hasselt University and East-Limburg Hospital (EudraCT B37120107805) and Comite de Etica de la Investigation Parc de salut Mar (No. 2018/8050/I), respectively. The Dutch MNP exposure cohort received ethics approval from the Medical Research Ethics Committee NedMec (NL81071.041.22). Adult participants in all cohorts provided informed consent for themselves and where applicable, for their children. An incidental finding policy is in place for all cohorts. All participants can withdraw from the study at any time, for any reason and without any consequences. Participants in the birth cohorts receive no compensation, whereas participants in the Dutch MNP exposure cohort receive a €25 (as of July 12, 2023 1 EUR=US $1.1135) gift card upon completion of the study.

## Results

In the first 3 years of this 5-year project (2021-2026), the following progress has been made. As of September 2024, data analysis has begun and results are expected to be published starting in 2025.

### In-Depth Characterization

We established a database consisting of infrared spectroscopy data measured on various polymers treated with different conditions; created a sample preprocessing workflow for measuring MNPs in complex human sample matrices, notably placenta; assessed the gaps in the current field of NP synthesis and fluorescent detection, synthesized NPs for 7 polymers including PVC, PS, PET, PA, PP, PMMA, and low-density PE using 2 fluorescent dyes; and summarized microspectroscopic techniques for measuring NPs [20].

### Scalable Exposure Assessment

We advanced critical operational procedures for scalable biomonitoring of MNPs in placenta tissue and blood, including developing high-throughput and robust sample preparation based upon microwave-assisted extraction. We developed a comprehensive MNPs library containing the Py-GC/HRMS signal fingerprints of 39 common polymers, alongside relevant information on their associated plastic additives. Sample analysis for the characterization of maternal and fetal MNP exposure and biological response in the BiSC and ENVIRONAGE cohorts and spatial analysis of whole placentas is ongoing.

### Toxicological Hazard Assessment Studies

We reviewed the models for assessing placental uptake, transport, and toxicity of MNPs [34] and optimized the BeWo/HUVEC coculture model, and the BeWo/HUVEC/H295R triculture. The Tier 3 placenta perfusion model and the Tier 4 zebrafish and mESD3 models have also been optimized. Screening and prioritization of MNPs in Tier 1 and Tier 2 are ongoing, and Tier 4 experiments with zebrafish are in progress.

### Epidemiological Studies

We recruited 110 participants for the Dutch MNP exposure cohort and collected blood, urine, and household dust samples. Sample collection from the birth cohorts for the epidemiological studies is complete; biobanked samples were selected from ENVIRONAGE, and de novo samples were collected from BiSC. Sample collection is complete for the spatial distribution study of MNPs in the placenta and ongoing for the study on the maternal-fetal transfer of MNPs. Follow-up of the birth cohorts is ongoing, and epidemiological analyses will proceed when MNP exposure estimates are available.

### Risk Assessment Framework

We completed the systematic evidence mapping and initial roadmap addressing risk assessment for MNPs in early life, highlighting the current challenges and limitations [78]. This will be updated when results from AURORA-associated studies are completed, and with emerging external research and gaps in knowledge and future research needs will be identified.

## Discussion

### Principal Findings

The AURORA project brings together experts with diverse knowledge, from producing plastics and measuring plastics and associated chemicals, to quantifying the biological impacts of nanoparticles and chemicals on reproductive health and development in vitro*,* in vivo*,* and in humans, as well as experts in risk assessment and risk communication to assess exposures to and health effects of MNPs during the critical period of early-life development. Advancements in detection, quantification, and characterization of MNPs are crucial for understanding human exposure to MNPs. AURORA will develop complementary low-throughput nondestructive particle-based approaches for characterization of MNP properties and high-throughput Py-GC/HRMS mass-based measurements for scalable exposure assessment. Toxicological testing will provide foundational insight into the toxicity of a diverse range of MNPs in test systems with varying complexity. The epidemiological investigations will provide the first extensive evaluation of maternal and prenatal MNP exposures. While developing and applying the tools and methodological workflows, a risk assessment framework specific to MNPs will be established.

MNPs have recently been detected in human placenta tissue, meconium, amniotic fluid, and breastmilk, and cumulative evidence from aquatic species indicates reproductive effects of MNPs [18,22,30,32,79]. In mice, maternal exposure to microplastics has been demonstrated to induce placental dysfunction and result in metabolic disorders in both the placenta and fetus. [80,81]. The diverse in vitro and in vivo human placental models in AURORA show potential for more comprehensively assessing the potential hazards of MNPs in utero. Currently, research investigating the potential risk MNPs may pose to early-life health in mammals remains limited [18,22,30,32,79]. In recent small-scale human studies, an inverse association between MNPs in amniotic fluid and gestational age was found, and placental MNPs were linked to intrauterine growth restriction [82,83]. Our results will enrich the understanding of MNP exposure in human tissues and provide insight for the first time about health effects of MNPs during early life. Understanding the exposure and hazard of MNPs in early life is a crucial first step toward determining whether public health actions are needed and informing the urgency of regulatory responses to MNPs. As the burden of plastic in the environment increases, more evidence about how MNPs affect human health is essential [4,84].

### Limitations

Given the complex nature of MNPs, there are challenging methodological advancements that need to be made during the project to accurately measure and evaluate early-life exposure to MNPs. Preliminary MNP measurements in human tissues suggest that exposure assessment is feasible; however, advancements in certain steps are necessary to develop workflows tailored to complex human matrices and ensure the reliability of exposure estimates. Additionally, the development of suitable test materials and workflows for toxicity testing, including dispersion protocols, requires significant methodological advancements. Another challenge we encounter is adequately addressing MNP contamination, which we do through the implementation of extensive QA/QC measures.

### Dissemination

The results of the study will be published in peer-reviewed journals and further disseminated, for example, through webinars, and scientific conferences. Analytical protocols will be made available to the research community whenever possible. We will also engage with stakeholders, including health care professionals, industry, civil society organizations, and policy makers, using a multichannel approach including the project website, newsletters, press releases, workshops, and social media. We will liaise with the European Commission and its Joint Research Centre to ensure that the findings are translated into policy. We will publish open access, including analytical scripts. Data and metadata will be stored on the eNanoMapper [85] data repository.

### Future Directions

AURORA supports the European Strategy for Plastics in a Circular Economy [86] by contributing to advancements of analytical methods for assessing thousands of chemicals, including potential unknown contaminants in future biodegradable and compostable plastics. The tiered approach for testing the toxicological effects of MNPs builds a framework for assessing the potential health impacts of measures taken under European policy. Aligned with the European Bioeconomy Strategy [87], AURORA addresses food and nutrition security, sustainable resource management, reduced reliance on nonrenewables, climate change mitigation, and European competitiveness. By providing novel information on health and safety risks of MNPs, AURORA will inform the development of safer plastics and bioalternatives for a circular economy.

Along with the gaps in health knowledge, policy and regulatory gaps must be addressed for systemic changes to be made. Harmonization of measurement techniques, exposure metrics, and terminology is essential to facilitate understanding between scientific and regulatory communities. Best practice is reporting both mass and particle counts, as will be done in AURORA [88]. Initiatives like an MNP-reporting guidelines checklist is a good example of moving MNP research forward in a valid, reproducible, and comparable way [89]. The harmonization of these key components will allow the newly generated research to collectively contribute to informing policy and control measures.

As the research on MNP and health is in its infancy, we acknowledge that not all open questions will be answered within AURORA or the CUSP cluster [38]. Therefore, in addition to the novel methods and tools and general framework for risk assessment of MNPs, we will develop an actionable risk assessment framework for MNP exposure in early life where the remaining knowledge gaps and priorities needed for comprehensively evaluating the impact of MNPs on early-life health are identified.

### Conclusions

As plastic pollution increases globally, it is imperative to understand the impact of MNPs on human health, particularly during vulnerable developmental stages such as early life. The contributions of the AURORA project are important for understanding how MNPs may influence health in early life. We will advance the research field by advancing characterization and quantification methods, providing novel toxicological and human health outcome data, and ultimately design a risk assessment framework to inform future MNP research.

## Appendix

Multimedia Appendix 1 Peer review.

## Abbreviations

AAH-HRMS: alkaline-assisted hydrolysis with high resolution mass spectroscopy
AURORA: Actionable eUropean ROadmap for early-life health Risk Assessment of micro- and nanoplastics
BeWo b30: human choriocarcinoma cell line
BiSC: Barcelona Life Study Cohort
ENVIRONAGE: ENVIRonmental influence ON early AGEing
FAIR data: findable, accessible, interoperable, reusable data
FaSTE-MPA: Fast, Single, Tissue Extraction for Multiplexed Plastic Analysis
H295R: human adenocarcinoma cell line
HUVEC: human umbilical vein endothelial cells
LC-HRMS: liquid chromatography with high resolution mass spectroscopy
mES-D3: mouse embryonic stem cell line
MNP: micro- and nanoplastic
MP: microplastic
NP: nanoplastic
OR: odds ratio
PA: polyamide
PE: polyethylene
PET: polyethylene terephthalate
PMMA: polymethyl methacrylate
PP: polypropylene
PS: polystyrene
PVC: polyvinyl chloride
Py-GC/HRMS: pyrolysis gas chromatography high-resolution mass spectroscopy
QA/QC: quality assurance and quality control

## Data Availability

Data sharing is not applicable to this article as no data sets were generated or analyzed during this study. Data from this project will be shared in future publications and data repositories including eNanoMapper.
