# Supplementary material for: Investigating Exposure and Hazards of Micro- and Nanoplastics During Pregnancy and Early Life (AURORA Project): Protocol for an Interdisciplinary Study
Source: JMIR Res Protoc. 2024 Oct 8;13:e63176. doi: 10.2196/63176 (PMC11496927; doi:10.2196/63176)
Supplement: Multimedia Appendix 1 [file resprot_v13i1e63176_app1.pdf]

Proposal Evaluation Form

EUROPEAN COMMISSION

Horizon 2020 - Research and Innovation Framework Programme

Evaluation Summary Report - Research and innovation actions

Call: H2020-SC1-2020-Single-Stage-RTD
Type of action: RIA
Proposal number: 964827
Proposal acronym: AURORA
Duration (months): 60
Proposal title: Actionable eUropean ROadmap for early-life health Risk Assessment of micro- and nanoplastics
Activity: SC1-BHC-36-2020

N.	Proposer name	Country	Total Cost	%	Grant Requested	%
1	UNIVERSITAIR MEDISCH CENTRUM UTRECHT	NL	1,125,415	18.77%	1,125,415	18.77%
2	FUNDACION PRIVADA INSTITUTO DE SALUD GLOBAL BARCELONA	ES	315,702.5	5.26%	315,702.5	5.26%
3	UNIVERSITEIT HASSELT	BE	459,908.75	7.67%	459,908.75	7.67%
4	ICAHN SCHOOL OF MEDICINE AT MOUNT SINAI	US	1,099,770	18.34%	1,099,770	18.34%
5	UNIVERSITEIT UTRECHT	NL	1,132,015	18.88%	1,132,015	18.88%
6	Masarykova univerzita	CZ	277,312.5	4.62%	277,312.5	4.62%
7	STICHTING VU	NL	569,977.5	9.51%	569,977.5	9.51%
8	ITA-SUOMEN YLIOPISTO	FI	340,070	5.67%	340,070	5.67%
9	Food Packaging Forum Foundation	CH	384,062.5	6.40%	384,062.5	6.40%
10	INSTITUTE OF OCCUPATIONAL MEDICINE	UK	213,550	3.56%	213,550	3.56%
11	CARL VON OSSIETZKY UNIVERSITAET OLDENBURG	DE	78,531.25	1.31%	78,531.25	1.31%
	Total:		5,996,315		5,996,315	

Abstract:
The scale of micro- and nanoplastic (MNP) pollution is becoming increasingly clear yet little is known about how this pollution impacts health. The AURORA project will deliver an actionable European roadmap for early-life health risk assessment of MNPs to support regulation of MNPs and the products and processes that generate secondary MNPs, and development of safer alternatives. We will focus on MNP exposures and toxicological and health effects during pregnancy, in utero, and in early life. These periods are critical for development and health later in life and are of heightened vulnerability to environmental insults. We have recently shown that MNPs are likely to cross the placental barrier in vitro and in vivo, underlying the urgent need to understand the impact of MNPs on reproductive and early-life health. AURORA will do so by significantly enhancing exposure assessment capabilities for measuring MNPs and MNP-associated chemicals (e.g. additives) in tissues relevant for early-life development (placenta, cord blood, amniotic fluid, meconium, fetal tissue). It will take a unique approach by combining in-depth characterization methods (microscopy and spectroscopy) and scalable methods (mass-spectrometry) to develop methods for both detailed and large-scale toxicological, exposure assessment, and epidemiological studies. This will be combined with a novel tiered-testing approach and epidemiological investigations to provide the first extensive evaluation of maternal and fetal MNP exposures and health perturbations, including placental function, immune-inflammatory responses, oxidative stress, accelerated aging, endocrine disruption, and child development. In the course of developing and applying the tools and methodological workflows of the AURORA research program, we will create a risk assessment framework specific to MNPs and identify the remaining knowledge gaps and priorities needed for comprehensively evaluating the impact of MNPs on early-life health.

Evaluation Summary Report

Evaluation Result

Total score: 14.50 (Threshold: 12)

Form information

SCORING

Scores must be in the range 0-5.

Interpretation of the score:

*0 The **proposal fails to address the criterion** or cannot be assessed due to missing or incomplete information.*

***1 Poor.** The criterion is inadequately addressed, or there are serious inherent weaknesses.*

***2 Fair.** The proposal broadly addresses the criterion, but there are significant weaknesses.*

***3 Good.** The proposal addresses the criterion well, but a number of shortcomings are present.*

***4 Very good.** The proposal addresses the criterion very well, but a small number of shortcomings are present.*

***5 Excellent.** The proposal successfully addresses all relevant aspects of the criterion.Any shortcomings are minor.*

Criterion 1 - Excellence

Score: **4.50** (Threshold: 4/5.00 , Weight: -)

The following aspects will be taken into account, to the extent that the proposed work corresponds to the topic description in the work programme:
Clarity and pertinence of the objectives
Soundness of the concept, and credibility of the proposed methodology
Extent that proposed work is beyond the state of the art, and demonstrates innovation potential (e.g. ground-breaking objectives, novel concepts and approaches, new products, services or business and organisational models)
Appropriate consideration of interdisciplinary approaches and, where relevant, use of stakeholder knowledge and gender dimension in research and innovation content

Overall, the objectives are pertinent, relevant, very clearly defined and in line with the call topic. The proposal presents a very carefully designed project, which focuses on the exposure to microplastics during pregnancy and early life, including an analysis of its toxic effects. The proposed concept is sound.
The proposed methods for assessment of bioaccumulation of microplastics in placental tissue are excellent. The toxicological methods proposed are of high quality and appropriate to meet the overall objectives of the proposal. There was some concern related to the contribution of background chemicals and microplastics, but this issue is adequately addressed in the risk assessment section.
The proposed study clearly goes beyond the state of the art, as there are very few studies identifying microplastics in tissues and current methods are poorly developed. The proposal delivers particularly strong innovations in human health hazard and risk assessment methodologies. The proposal is very ambitious but credible, as it is based on preliminary work of the consortium members, indicating that microplastic particles are actively transported in tissues during perfusion experiments. The proposed work has an excellent chance of identifying particles in tissues if they are present. This will be a significant advancement of the current state of knowledge. The proposed analysis and toxicology assessment is adequately complemented by cohort studies and a proof-of-principle intervention study. The innovative perfusion experiments are useful alternatives to animal experimentation. Therefore, the proposal shows high innovation potential and goes significantly beyond the state of the art.
The approach proposed is interdisciplinary as it brings together a wide range of relevant disciplines. The cross-cutting issue of gender is appropriately considered in the context of the research. Socio-economic factors will be appropriately considered in epidemiologic studies as social factors related to microplastic exposure will be studied.
The different stages of the project are based on interdisciplinary work across the consortium. The inclusion of a source of stakeholder knowledge on plastics and their composition is appropriate. The external stakeholder panel will be built of interdisciplinary researchers and consulted throughout the project to ensure that their perspectives are taken into account in the design and implementation of the research.

Criterion 2 - Impact

Score: **5.00** (Threshold: 4/5.00 , Weight: -)

The following aspects will be taken into account:
The extent to which the outputs of the project would contribute to each of the expected impacts mentioned in the work programme under the relevant topic
Any substantial impacts not mentioned in the work programme, that would enhance innovation capacity, create new market opportunities, strengthen competitiveness and growth of companies, address issues related to climate change or the environment, or bring other important benefits for society
Quality of the proposed measures to:
- exploit and disseminate the project results (including management of IPR), and to manage research data where relevant
- communicate the project activities to different target audiences

Overall, the proposal contributes in an excellent way to the expected impacts of the call requirements. It addresses the great majority of expected impacts mentioned in the call topic while providing a more in-depth study of reproductive effects, which are potentially more sensitive to exposures to micro- and nanoplastics (MNPs). It will make an excellent contribution to understanding the health impact of MNPs in early life and will provide data on current birth cohorts to potentially identify long-term impacts. The proposal will contribute very well to the health-relevant aims of the European Strategy for Plastics by supporting the development and exploitation of innovative strategies. The outcomes of the project have the potential to make a significant impact on methods for analysis and, subsequently, on knowledge of occurrence and exposure to MNPs. They will also increase the knowledge of uptake and toxicity and, possibly, bioaccumulation of MNPs.
The proposal contributes very well to the cross-cutting issue of open innovation and will encourage co-creation by providing access to very valuable biological samples, in particular samples from the placental perfusion and 3D culture models. The proposals for analysis of exposure and effects will provide a platform for analysis of effects in other vulnerable groups in future studies, and thereby a solid foundation for progress. The proposal does not explicitly describe its contribution to the Social Sciences and Humanities cross-cutting issue, although social factors are considered in the cohort studies.
Dissemination plans are appropriate and describe very well the targeted activities. The external stakeholder panel has already been planned in detail and presented with specific contacts named. The audiences for dissemination activities are relevant and appropriate. Communication is well-balanced between scientific, political and public (social media, website, news, etc.) arenas.
The project will closely collaborate with the European Commission Joint Research Centre (JRC) to provide added value regarding a number of aspects.
Plans for exploitation of the research results are well developed as well. All project partners are experienced in participating in EU projects, which is reflected in well-thought out communication and dissemination plans.

Criterion 3 - Quality and efficiency of the implementation

Score: **5.00** (Threshold: 3/5.00 , Weight: -)

The following aspects will be taken into account:
Quality and effectiveness of the work plan, including extent to which the resources assigned to work packages are in line with their objectives and deliverables
Appropriateness of the management structures and procedures, including risk and innovation management
Complementarity of the participants and extent to which the consortium as a whole brings together the necessary expertise
Appropriateness of the allocation of tasks, ensuring that all participants have a valid role and adequate resources in the project to fulfil that role

The overall work plan is very clear and effective. Work packages are clearly and appropriately scheduled. The allocation of resources, including person-months to work packages, is also clear and well planned. The tasks within the work packages and milestones are very well spaced timewise. The deliverables are appropriately spaced throughout the project. Moreover, the deliverables proposed are well defined and

provide useful results and resources. Overall, the work plan appears to be very well thought out with appropriate resources allocated to each task and with a good prospect of successful completion of the proposed work.
The management structure is clearly presented, and roles of the various actors are clearly defined. The general assembly and executive board are well planned and the description of how they will work with external stakeholders and the scientific advisory panel is coherent and appropriate. The consortium proposes to build on existing resources and studies, which will be an efficient way of delivering results. A wide range of relevant and adequate risks are considered, including technical risks and potential COVID-19 pandemic related risks.
The complementarity of the proposed consortium is excellent and it brings together relevant expertise. The allocation of tasks is appropriate, and all participants have a valid role and adequate resources to fulfil their roles. The different participants contribute to each task to differing extents but the overall level of interdisciplinary participation is high, and the different participants do complement each other with related skills. The proposal will join activities with other selected projects under topics supporting the EU Plastics Strategy and Bioeconomy Strategy.
Overall, the information provided supports the view that i) the participants will be able to carry out the work in which they are participating to an adequate level; ii) the consortium certainly has the range and depth of expertise required; and iii) the overall resource allocation is appropriate for the proposed research and other activities.

Scope of the proposal

Status: **Yes**

Comments (in case the proposal is out of scope)

Not provided

Operational Capacity

Status: **Operational Capacity: Yes**

If No, please list the concerned partner(s), the reasons for the rejection, and the requested amount.

Not provided

Exceptional funding of third country participants/international organisations

A third country participant/international organisation not listed in General Annex A to the Main Work Programme may exceptionally receive funding if their participation is essential for carrying out the project (for instance due to outstanding expertise, access to unique know-how, access to research infrastructure, access to particular geographical environments, possibility to involve key partners in emerging markets, access to data, etc.). (For more information, see the Online Manual)

Based on the information provided in the proposal, we consider that the following participant(s)/international organisation(s) that requested funding should exceptionally be funded:
(Please list the Name and acronym of the applicant, Reasons for exceptional funding and the Requested grant amount.)

Not provided

Based on the information provided in the proposal, we consider that the following participant(s)/international organisation(s) that requested funding should NOT be funded:
(Please list the Name and acronym of the applicant, Reasons for exceptional funding and the Requested grant amount.)

Not provided

Use of human embryonic stem cells (hESC)

Status: **No**

If yes, please state whether the use of hESC is, or is not, in your opinion, necessary to achieve the scientific objectives of the proposal and the reasons why. Alternatively, please state if it cannot be assessed whether the use of hESC is necessary or not because of a lack of information.

Not provided

FUNDING & TENDERS PORTAL E-RECEIPT

Digitally sealed by the European Commission

Date: 2020.10.20 08:52:48 CEST

This electronic receipt is a digitally signed version of the document submitted by your organisation. Both the content of the document and a set of metadata have been digitally sealed.

This digital signature mechanism, using a public-private key pair mechanism, uniquely binds this eReceipt to the modules of the Funding & Tenders Portal of the European Commission, to the transaction for which it was generated and ensures its full integrity. Therefore a complete digitally signed trail of the transaction is available both for your organisation and for the issuer of the eReceipt.

Any attempt to modify the content will lead to a break of the integrity of the electronic signature, which can be verified at any time by clicking on the eReceipt validation symbol.

More info about eReceipts can be found in the FAQ page of the Funding & Tenders Portal.

(https://ec.europa.eu/info/funding-tenders/opportunities/portal/screen/support/faq)
